# *Yersinia* infection tools—characterization of structure and function of adhesins

**DOI:** 10.3389/fcimb.2012.00169

**Published:** 2013-01-08

**Authors:** Kornelia M. Mikula, Robert Kolodziejczyk, Adrian Goldman

**Affiliations:** ^1^Macromolecular X-Ray Crystallography Group, Structural Biology and Biophysics, Institute of Biotechnology, University of HelsinkiHelsinki, Finland; ^2^The National Doctoral Program in Informational and Structural Biology, Åbo AcademyTurku, Finland

**Keywords:** adhesins, bacterial, *Yersinia enterocolitica*, *Yersinia pseudotuberculosis*, *Yersinia pestis*, outer membrane proteins, X-ray structure, structure–function relationship

## Abstract

Among the seventeen species of the Gram-negative genus *Yersinia*, three have been shown to be virulent and pathogenic to humans and animals—*Y. enterocolitica*, *Y. pseudotuberculosis*, and *Y. pestis*. In order to be so, they are armoured with various factors that help them adhere to tissues and organelles, cross the cellular barrier and escape the immune system during host invasion. The group of proteins that mediate pathogen–host interactions constitute adhesins. Invasin, Ail, YadA, YadB, YadC, Pla, and pH 6 antigen belong to the most prominent and best-known *Yersinia* adhesins. They act at different times and stages of infection complementing each other by their ability to bind a variety of host molecules such as collagen, fibronectin, laminin, β1 integrins, and complement regulators. All the proteins are anchored in the bacterial outer membrane (OM), often forming rod-like or fimbrial-like structures that protrude to the extracellular milieu. Structural studies have shown that the anchor region forms a β-barrel composed of 8, 10, or 12 antiparallel β-strands. Depending on the protein, the extracellular part can be composed of several domains belonging to the immunoglobulin fold superfamily, or form a coiled-coil structure with globular head domain at the end, or just constitute several loops connecting individual β-strands in the β-barrel. Those extracellular regions define the activity of each adhesin. This review focuses on the structure and function of these important molecules, and their role in pathogenesis.

## Introduction

*Yersiniae* belong to the Enterobacteriaceae family; they are Gram-negative, facultative anaerobes. Seventeen different species of *Yersinia* genus have so far been reported, of which three have been shown to be pathogenic to humans and animals. These are the enteropathogens *Y. enterocolitica* and *Y. pseudotuberculosis*, and one zoonotic pathogen *Y. pestis*. Pathogenicity is correlated mostly with 70-kb virulence plasmid pYV carried by all three of them and with additional chromosomally encoded proteins (Cornelis, 1994). Other *Yersinia* species are either avirulent or their pathogenicity has not been reported.

**Figure 1:**
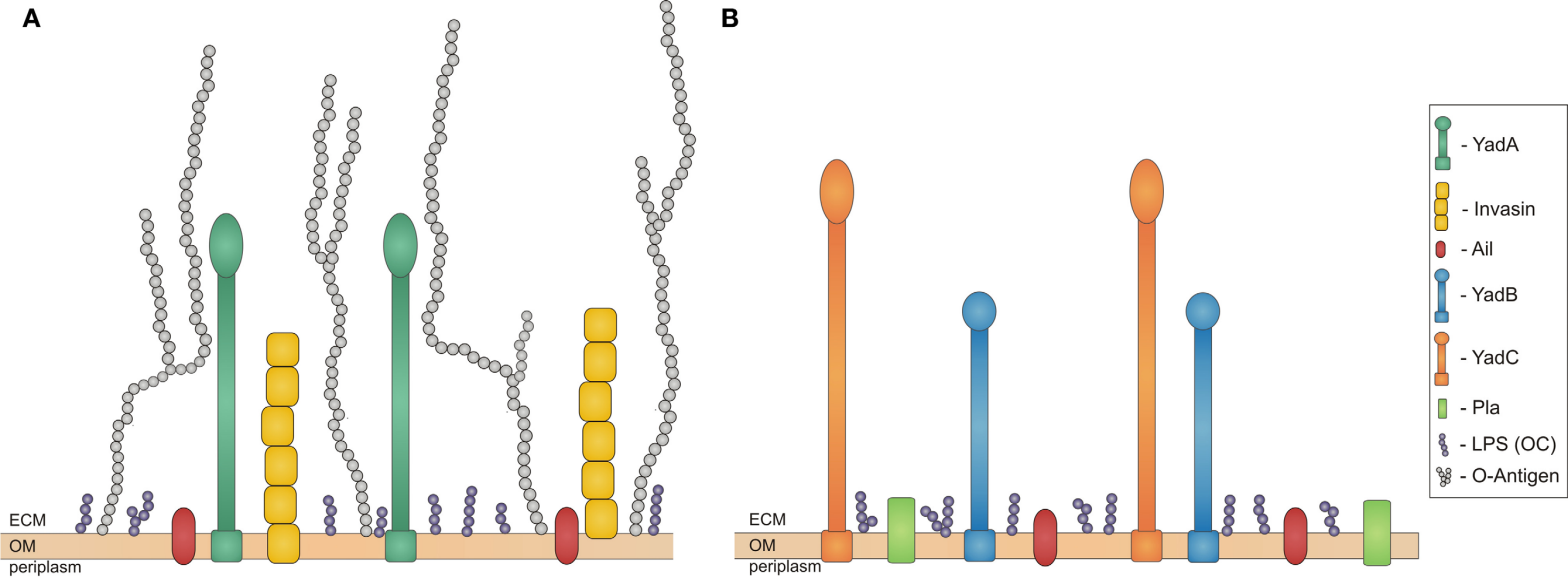
**Schematic overview of proteins expressed in *Yersiniae* outer membrane during infection.** Bacterial outer membrane (OM) with outer core of LPS (OC) in purple and adhesins expressed at different stages of infection. **(A)** Adhesisn of *Y. enterocolitica* and *Y. pseudotuberculosis*: invasin in yellow, YadA in dark green, Ail in red, and O-Antigen in light grey; **(B)** Adhesins of *Y. pestis*: Pla in green, YadB in blue, YadC in orange, Ail in red. ECM stands for extracellular matrix. All the molecules are on approximately the same scale.

*Y. enterocolitica* and *Y. pseudotuberculosis* are responsible for a wide range of diseases from mild diarrhoea, enterocolitis, septica, and mesenteric lymphadenitis to reactive arthritis and iritis (Cover and Aber, 1989). They are transmitted by ingestion of contaminated food. The most frequent outbreaks of *Y. enterocolitica* have had their origin in infected, undercooked pork meat, but bacteria have also been found in other mammalian hosts (Bottone, 1997). The most common reservoirs for *Y. pseudotuberculosis*, on the other hand, have been reported to be carrots and lettuce (Jalava et al., 2006). Immediately after oral uptake of contaminated food or water (Mazigh et al., 1984), bacteria traverse through the gastrointestinal tract until they reach the terminal ileum. At this point bacteria already have present on their surface the outer membrane (OM) protein invasin, which is expressed in stationary phase at low temperatures (e.g., in stored food) (Pepe and Miller, 1993). It plays a crucial role during the first phases of infection by facilitating efficient translocation across the intestinal epithelial barrier. During this phase, a second protein, Ail, is also important (Figure 1A). Bacteria traverse the epithelial barrier through the M cells (microfold cells) that are associated with Payer's patches (Grassl et al., 2003).

After translocation to the basolateral side of the Peyer's patches, invasin binds to β_1_ integrins, which induce production of chemokines like IL-8. In the Payer's patches, bacteria replicate and express another adhesin, YadA (Figure 1A), which protects bacteria against phagocytosis of recruited polymorphonuclear leukocytes (PMN) and monocytes, as well as downregulates expression of invasin. PMN and monocytes may lead to tissue disruption and bacterial transport to gut associated lymphoid tissues. YadA and Ail enable bacterial dissemination to mesenteric lymph nodes by protecting against the host immune system. Moreover, YadA facilitates adhesion to collagen, which is crucial for *Y. enterocolitica* in causing reactive arthritis, a sterile inflammation of the joints. This iritis and erythema nodosum are post-infection sequelae of *Y. enterocolitica* mediated by YadA (Cover and Aber, 1989). In more severe infections bacteria can further colonize other organs like liver, spleen, kidney, or lungs, but infection is usually self-limiting (Grassl et al., 2003).

The third human pathogenic *Yersnia* species, *Y. pestis*, evolved around 1500–20,000 years ago from *Y. pseudotuberculosis* by lateral gene transfer and gene inactivation (Achtman et al., 1999). *Y. pestis* lost non-essential housekeeping genes, has inactivated genes encoding for proteins needed for intestinal pathogenesis, like invasin and YadA, although it is not known if inactivation of *yadA* and *inv* increased *Y. pestis* virulence (Achtman et al., 1999). The key step in the evolution of *Y. pestis* was the acquisition of the pFra plasmid. This, together with the ability to express chromosomally encoded proteins that *Y. pseudotuberculosis* was not enables *Y. pestis* to be transmitted by fleas from one mammalian host to another. *Y. pestis* expresses unique proteins associated with virulence, Pla (Achtman et al., 1999) and the recently discovered YadA and YadC (Figure 1B) (Forman et al., 2008). *Y. pestis also* expresses Ail and pH 6 antigens that are present in other *Yersinia* species (Figure 1B).

*Y. pestis* is the most virulent and invasive of the three species, causing highly fatal pneumonic, bubonic, and septicemic plague (Perry and Fetherston, 1997). Pneumonic plague is the least common but most deadly form, progressing very rapidly from flu-like symptoms to overwhelming pneumonia. It spreads by inhalation of respiratory droplets during contact with the infected person (Perry and Fetherston, 1997). It may also occur as a complication after bubonic or septicaemic plague. If treatment is not started during the first 24 h after the first symptoms, it is usually fatal within 48 h (Felek et al., 2010). Bubonic plague (the most common form) occurs a few days after an infected fleabite or wound exposure to contaminated material. It causes swollenness and tenderness of lymph nodes, as well as gastrointestinal complaints. Very often secondary plague septicemia or bacteremia can occur, which is also highly fatal if untreated (40–60% mortality rates) (Perry and Fetherston, 1997). Finally, primary septicemic plague is present mainly in the bloodstream. Infection occurs by fleabites, contact with infectious material via open wounds or spread from the lymphatic system as a result of advanced stages of bubonic plague (Felek et al., 2010). The mortality rate of this forms 30–50% if left untreated (Perry and Fetherston, 1997).

In bubonic plague, *Y. pestis* travels from the initial site of infection to lymph nodes, most likely inside macrophages. When bacteria reach the lymph nodes, they escape from the macrophages and start to grow extracellularly to high numbers, leading to the formation of bubos (swollen lymph nodes). Bacteria resistant to phagocytosis spread into the bloodstream causing septicaemic plague. Moreover, infection can continue and bacteria further colonize blood, liver, spleen, or even lungs, which leads to secondary pneumonic plague (Perry and Fetherston, 1997). Expression of Pla enables *Y. pestis* to disseminate from the initial side of infection to lymph nodes and travel in the bloodstream. Pla also facilitates serum resistance. Moreover, while bacteria travel in the macrophages, expression of pH 6 antigen, another adhesin, is induced. This prevents plague bacteria from phagocytosis by those macrophages and after escape, from later phagocytosis (Perry and Fetherston, 1997). Expression of Ail helps bacteria to survive in blood as it is involved in serum resistance and mediates adhesion to epithelial cells and extracellular matrix (ECM) proteins (Miller et al., 2001). Of all the adhesins expressed by *Y. pestis*, Ail is the most important for Yop delivery (*Yersinia* outer proteins, secreted by the *Yersinia* III type secretion system) (Felek et al., 2010).

In this review, we focus on structural and functional aspects of the adhesins Invasin, YadA, YadB, YadC, Ail, Pla, and pH 6 antigen (Tables 1, 2), which are expressed during host invasion by *Yersinia* species.

**Table 1:** **Summary of functions of *Yersinia* adhesins**.

**Protein**	**Function**	**Organism**	**References**
**CHROMOSOMALLY ENCODED PROTEINS**			
Invasin	Invasion of epithelial cells	*Ye*^a^, *Yp*^a^	Isberg et al., 1987
	β_1_ integrin binding	*Ye, Yp*	Clark et al., 1998
	Induction of cytokine production	*Ye, Yp*	Grassl et al., 2003
YadB, YadC	Invasion of epithelial cells	*Ype*^a^	Forman et al., 2008
	Not known	*Yp*	Forman et al., 2008
Ail	Adhesion to epithelial cells	*Ye, Ype*	Miller et al., 2001
	Binding to laminin and fibronectin	*Ye, Ype*	Yamashita et al., 2011
	Serum resistance	*Ye, Yp, Ype*	Biedzka-Sarek et al., 2008b
	Yop delivery^b^	*Ye, Yp, Ype*	Felek et al., 2010
pH 6	Resistance to phagocytosis	*Ye, Ype*	Yang et al., 1996; Huang and Lindler, 2004
	Escape from macrophages	*Ype*	Lindler and Tall, 1993
	Haemagglutination	*Yp, Ype*	Yang et al., 1996
	Interaction with lipoproteins	*Ype*	Makoveichuk et al., 2003
	Interaction with Fc of IgG	*Ype*	Zav'yalov et al., 1996
	Yop delivery	*Ype*	Felek et al., 2010
	Tissue adhesion	*Yp*	Yang et al., 1996
	Adhesion	*Ye*	Yang et al., 1996
**PLASMID ENCODED PROTEINS**			
YadA	Essential for virulence	*Ye*	Roggenkamp et al., 1995
	Invasion of epithelial cells	*Yp*	Eitel and Dersch, 2002
	Binding to ECM molecules collagen, fibronectin, and laminin	*Ye, Yp*	Schulze-Koops et al., 1992; Flügel et al., 1994; Heise and Dersch, 2006
	Adhesion to epithelial cells, neutrophils, and macrophages	*Ye, Yp*	Heesemann et al., 1987; Roggenkamp et al., 1996
	Serum resistance	*Ye, Yp*	Lambris et al., 2008
	Autoagglutination	*Ye, Yp*	Hoiczyk et al., 2000
	Yop delivery	*Ye, Yp*	Visser et al., 1995
Pla	Plasminogen activation	*Ype*	Beesley et al., 1967
	Adherence and invasion to epithelial cells	*Ype*	Sodeinde et al., 1992
	Degradation of laminin and fibrin	*Ype*	Haiko et al., 2009
	Serum resistance	*Ype*	Sodeinde et al., 1992
	Yop delivery	*Ype*	Felek et al., 2010

a
Abbreviations Yp Yersinia pseudotuberculosis Ye Yersinia entercolitica Ype Yersinia pestis.

b *Indirect, via laminin and fibronectin binding*.

**Table 2:** **Summary of structures of *Yersinia* adhesins discussed**.

**Protein**	**Region**	**PDB**	**Localization**	**Organism**	**References**
Invasin	D1-D5 domains	1CWV	extracellular	*Yp*^a^	Hamburger et al., 1999
	β-barrel	4E1T	outer membrane	*Yp*	Fairman et al., 2012
YadA	Head + neck	1P9H	extracellular	*Ye*^a^	Nummelin et al., 2004
Ail	β-barrel	3QRA	outer membrane	*Ype*^a^	Yamashita et al., 2011
Pla	β-barrel	2X55	outer membrane	*Ype*	Eren et al., 2010

a
Abbreviations Yp Yersinia pseudotuberculosis Ye Yersinia entercolitica Ype Yersinia pestis.

## Invasin–the first adhesin expressed during invasion of enteropathogenic *yersinia*

Invasin is an adhesin expressed by enteropathogenic (EPEC) species of *Yersinia, Y. enterocolitica*, and *Y. pseudotuberculosis* (Isberg et al., 1987). It acts during the first phase of infection, and is responsible for initial colonization and internalization with host cells.

Invasin is chromosomally encoded by the *inv* gen, which is maximally expressed at 25°C, pH 8 or at 37°C, pH 5.5 but poorly at 37°C, pH 8. This indicates that invasin is expressed prior to oral uptake (i.e., in stored food), which may be beneficial for rapid transcytosis through the epithelial layer, or in the intestinal tissue (Grassl et al., 2003; Uliczka et al., 2011). Difference in expression efficiency of invasin depends on *Y. enterocolitica* serotype and the regulatory factors present in the serotypes. Invasin expression is repressed at 37°C in the O:8 and O:9 serotypes by rapid degradation of the *invA* activator RovA (transcriptional activator), and silencing mediated by negative regulator H-NS (nucleoid structuring protein), which forms higher order complexes in a concentration-dependent manner and causes gene silencing (Wyborn et al., 2004; Uliczka et al., 2011). Constitutive expression of invasin at 25°C, as well as at 37°C in the O:3 serotype, results from insertion of the IS1667 element into the regulatory region of the *invA* gene. This disrupts the inhibitory region that binds H-NS (Uliczka et al., 2011). Moreover, it has been shown that the single amino acid substitution P98S increases the thermostability of RovA, leading to higher expression of invasin (Uliczka et al., 2011). The nature of this stabilization is uncertain as no major structural changes have been observed, but it leads to greatly decreased proteolysis of RovA (Uliczka et al., 2011). Nonetheless, despite the large amount of invasin expressed at 25 and 37°C by the O:3 strain, cell invasion is significantly reduced or does not occur at all for bacteria pregrown at 25°C (Bialas et al., 2012). This appears to be because high expression of the O-antigen in the LPS (lipopolysaccharide) creates steric hindrance that prevents interactions between invasin and the host cell surface whereas at 37°C expression of O-antigen is repressed allowing better access of invasin to host cells. This is unlike other *Y. enterocolitica and Y. pseudotubrculosis* serotypes, where the highest level of invasin expression occurs when they are cultured at moderate temperatures (Uliczka et al., 2011).

**Figure 2:**
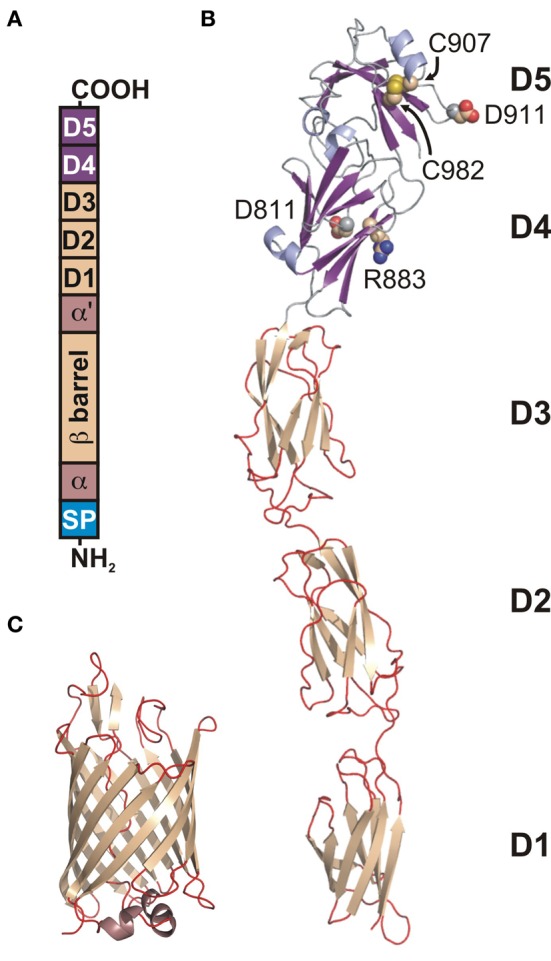
**Modular architecture of the *Y. pseudotuberculosis* invasin. (A)** A schematic topology model of preprotein. From N-terminus: (SP) signal peptide, (a) periplasmic hydrophilic α-domain, (β-barrel) OM embedded β-barrel domain, (α') periplasmic hydrophilic α'-domain, (D1–D4) extracellular Ig-like domains, (D5) extracellular distal domain of the C-type lectin-like fold. D4 and D5 form a functional integrin binding module. **(B)** Ribbon diagram of the structure of D1–D5 (1CWV). Residues D811, R883, C907, D911, and C982 (space-filling representation) are implicated in integrin binding. **(C)** Ribbon diagram of the β-barrel structure (4E1T).

Invasins (about 92 kDa) are closely related sequentially and structurally to the intimins, OM proteins from the EPEC and enterohemorrhagic (EHEC) *E. coli* strains. A topology model (Tsai et al., 2010) suggested that the invasin/intimin family has a conserved modular architecture, composed of: (1) signal sequence, (2) hydrophilic α-domain, (3) β-barrel domain, (4) hydrophilic α′-domain, and (5) extracellular domain (Figure 2A). The structures of the invasin extracellular domain (1CWV) (Figure 2B) (Hamburger et al., 1999) and β-barrel (PDB: 4E1T) (Figure 2C) (Fairman et al., 2012) reflect a modular architecture. The signal sequence allows translocation through the inner membrane (IM) via the Sec secretion mechanism and is cleaved off afterwards. There are two hydrophilic α-domains that reside in the periplasm and are separated in sequence by the β-barrel.

By having a β-barrel at the N-terminus and the extracellular domain at the C-terminus (Figure 1A), Invasin has an inverse arrangement to the classical autotransporter (AT) system (type V secretion system), and so has been proposed to constitute a new type Ve AT system (Leo et al., 2012). The AT system requires autonomous passage of the extracellular “passenger” domain from the periplasm to the extracellular space without an external energy source such as ATP or a proton gradient (Thanassi et al., 2005). This most likely happens through the β-barrel (transporter domain) that forms a pore in the OM, and which works also as an anchor for the passenger domain after it has been transported to the extracellular space. The proper folding of ATs occurs with the help of the multicomponent BAM machinery and chaperones (Leyton et al., 2012), but the precise mechanism still remains unknown.

The barrel, similarly to AT β domains, is composed of 12 β-strands arranged in an antiparallel fashion. The barrel pore is filled with a linker, which in contrast to transporter domains of AT is not α-helical but instead adopts an extended conformation. In addition, the periplasmic α-helix connecting the transporter domain with a linker to the passenger domain does not occur in ATs (Figure 2C).

The passenger domain has a rod-like shape with overall dimensions of 180 × 30 × 30 Å. It is composed of three to four mainly β domains (D1–D4) terminated with an α +β domain D5. D1–D4 form bacterial immunoglobulin-like (BIG) domains, from which D1 belongs to the I2 set of the immunoglobulin superfamily, D2 and D3 belong to the I1 set, and D4 to the C1 set. D5 has a folding topology related to the C-type lectin-like domains (Figure 2B) (Hamburger et al., 1999). D2 is not present in invasin from *Y. enterocolitica*. The D4–D5 domains form a functional module that is sufficient for binding to integrins with an affinity about 100 times higher than fibronectin (Van Nhieu and Isberg, 1991; Van Nhieu et al., 1996) and that is very important for penetration of mammalian cells. This higher affinity may be caused by the fact that the integrin-binding surface is larger in invasin than in fibronectin. This enables pathogenic bacteria to compete for binding with host proteins, exploiting a host receptor for their own purposes. Two aspartate residues, Asp811 and Asp911, together with a disulphide bond between Cys907 and Cys982 (numbering according to *Y. pseudotuberculosis*) (Figure 2B) are important for integrin binding (Leong et al., 1993; Saltman et al., 1996). The S-S bond is presumably required for correct folding. The spatial arrangement of Asp811 and Asp911 (Figure 2B) is very similar to the Asp present in the RGD motif and synergy region in fibronectins. In both proteins the distance between Asp residues is 32 Å and they both contain arginine residue (Arg883 in invasin and Arg1379 in fibronectin) that is located nearby at the same distance from Asp911 and its equivalent in fibronectin. This overall similarity in the relative positions of Asp and Arg residues enables invasin to share common integrin-binding features with fibronectin (Hamburger et al., 1999).

During the first phase of infection, immediately after oral uptake of *Yersinia*, invasin initiates the internalization of bacteria to the epithelial cells of the small intestine, in particular to M cells, and maintains the invasion of the Payer's patches (Jepson and Clark, 1998). This process occurs due to binding of invasin to β_1_ integrins including α_3_β_1_, α_4_β_1_, α_5_β_1_, α_6_β_1_, and α_v_β_1_ present on the host cells (Clark et al., 1998). However, the critical factor for adhesion and uptake of *Yersinia* cells is the density of invasin particles present on the *Yersinia* OM and the density of β_1_ receptors on the host cells. Both have to be high, otherwise just adhesion, but not internalization, occurs (Dersch and Isberg, 1999; Isberg et al., 2000). Binding of invasin to integrins results in formation of integrin clusters, which triggers remodeling of the actin cytoskeleton and leads to internalization of the bacteria to the epithelial cells. This is called the “zipper” invasion mechanism (Grassl et al., 2003). Internalization allows delivery of Yops, which are required for *Yersinia* virulence (reviewed in Viboud and Bliska, 2005) to the host cell. This is followed by activation of two different signaling cascades. The first one mainly involves different phosphatases, one of which is the tyrosine phosphatase YopH. YopH disrupts focal adhesion complexes by dephosporylating proteins that comprise them, like FAK, FYB, paxillin, and p130Cas (Grassl et al., 2003). In the second signaling pathway, one can observe activation of NF-κB, various proinflammatory cytokines, including interleukin-8 (IL-8), IL-1α, IL-1β, tumour necrosis factor-α (TNF-α), and others, resulting in activation of host defence (Kampik et al., 2000).

## YadA—the multifunctional adhesin of enteropathogenic *yersinia*

YadA is the most studied and best described trimeric AT protein. Most of the activities of *Yersinia* now attributed to YadA were described before YadA itself. These are: establishing infection, adhesion to ECM molecules and others (Emödy et al., 1989; Tertti et al., 1992), autoagglutination (Skurnik et al., 1984), serum resistance (Balligand et al., 1985), phagocytosis resistance, and invasion (Heesemann et al., 1987).

YadA is encoded on pYV, the virulence plasmid, and expressed as a virulence factor by EPEC *Y. enterocolitica* and *Y. pseudotuberculosis* immediately after they cross the intestinal mucosa. Protein expression is regulated by temperature and induced when the bacterium is exposed to temperatures of +37°C (Zaleska et al., 1985; Skurnik and Toivanen, 1992). YadA is essential for virulence in *Y. enterocolitica*, (Roggenkamp et al., 1995), but not in *Y. pseudotuberculosis* (Han and Miller, 1997). Additionally, the *yadA* gene is present in the third human pathogen *Yersinia* species, *Y. pestis*, but as a pseudogene, due to a frame shift caused by single nucleotide deletion (Skurnik and Wolf-Watz, 1989). However, Forman et al. (2008) recently discovered and characterized two chromosomal genes *yadB* and *yadC* similar to *yadA* (see below).

**Figure 3:**
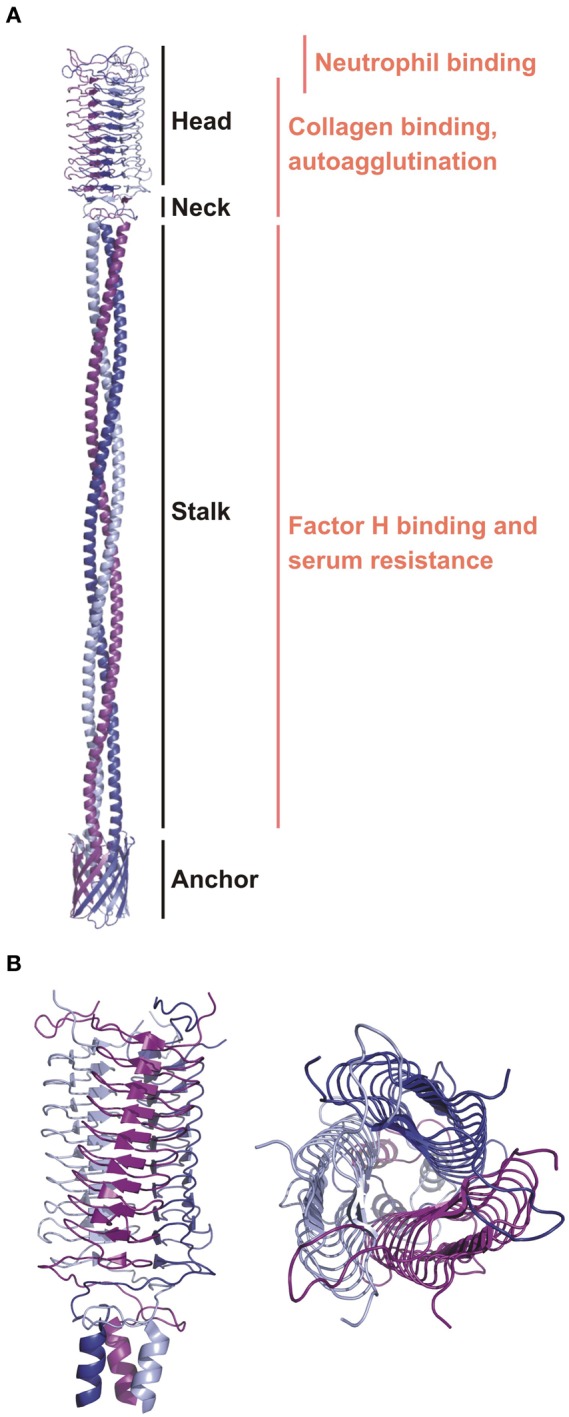
**The ribbon diagrams of YadA. (A)** A general topology model of YadA created based on the experimental structure of the head region and modeled stalk and anchor regions (Koretke et al., 2006). Modular organization and binding regions for different ligands are annotated. **(B)** The crystal structure of the YadA head domain from *Y. enterocolitica* (1P9H). Left panel is a side view showing the LPBR fold. The top view on the right panel presents trimeric organization of YadA.

Parts of the collagen-binding domain of YadA (1P9H) (Nummelin et al., 2004) and stalk fragments (3H7X, 3H7Z, 3LT6, 3LT7) (Alvarez et al., 2010) are available as experimental models, allowing the construction of a complete model of the YadA passenger domain (Figure 3A). YadA constitutes a prototypical non-fimbrial adhesin for which a theoretical model supported by structural data and sequence similarity analysis was created (Koretke et al., 2006). This model is characteristic of the overall basic architecture of trimeric autotransporter adhesins (TAA).

TAAs are obligate homotrimeric proteins with a common modular organization analogous to classical ATs. They consist of an N-terminal extracellular (passenger) domain and a C-terminal β-barrel (translocation domain) anchored in the OM. In TAAs the passenger domain is highly variable in sequence and size (200–3000 amino acid residues). The exact reason for the huge variability is not known, but Linke et al. (2006) suggest that this is because of the “red queen” principle (Van Valen, 1973): organisms that co-evolve, such as a pathogen and a host, must change continuously or else their evolutionary fitness will decrease. For a pathogen to remain equally virulent as when it first acquired a new host, its adhesins must evolve all the time: the TAAs solve this problem elegantly by being long and repetitive, leading to facile recombination.

The passenger domains contain three main regions: stalk, connector, and head (Figure 3A) (Szczesny and Lupas, 2008), which are diverse and appear in repetitively in different combinations. Currently the most complete structure of a TAA passenger domain is of EibD (Leo et al., 2011), which binds IgG Fc and IgA Fc in a non-immune manner, presumably to help in avoiding attack by the classical pathway of complement. The translocation domain is much more conserved. It is composed of 12 antiparallel β-strands. Such organization requires that each TAA subunit contribute four strands to make one third of the 12-strand barrel. As a result, the linker region between the translocator and passenger domain, a short helix partly embedded in the barrel, is triplicated and fills the pore.

YadA varies in length between 422 and 455 amino acid residues (El Tahir and Skurnik, 2001). It forms a lollipop-like projection of about 23 nm in length from the surface of the bacteria (Hoiczyk et al., 2000). This projection consists of a stem-like segment of ~18 nm (stalk) and bulky head of ~5 nm. The structure of the YadA head domain from *Y. enterocolitica* was the first to show the left-handed parallel β-roll (LPBR) fold (Figure 3B) (Nummelin et al., 2004). Short β-strands form layers that make the LPBR, which has a right-handed superhelical twist. It is ~53 Å long and ~37 Å wide, has an extremely hydrophobic core, and is stabilized at both ends where the coils tie the head's subunits like a lock nut. The stalk starts as is characteristic of all YadA-type TAAs as a right-handed coiled coil. There are ten 15-residue right-handed repeats, which deform at the bottom to left-handed canonical coiled coil heptads. These continue into the conserved anchor domain formed by four transmembrane β-strands per monomer (Figure 3A) (Koretke et al., 2006).

The most important activity of *Y. enterocolitica* YadA (YeYadA) is adhesion. It binds fibrillar collagen types I, II, III, V (Emödy et al., 1989; Schulze-Koops et al., 1992), and XI, and the network-forming collagen type IV (Flügel et al., 1994). On the cell, these interactions are very stable, tolerating extremes of pH and temperature, and are resistant to proteases and urea (Emödy et al., 1989). Binding of collagen type I by some *Y. enterocolitica* strains is stable at pH values from 5.0 to 10.0, and after heating for 20 min at 80°C at pH 7. To reduce binding of collagen I by 70% requires boiling for 1 h. The collagen-*Ye* interaction is resistant to proteolytic enzymes like proteinase K, papain, and trypsin. In addition, binding of collagen type I to YadA on the surface of *Y. enterocolitica* is not affected at all by 1 M urea; binding decreases to 32% in 3 M urea; and is still detectable (3%) in 6 M urea (Emödy et al., 1989).

It has been shown that the trimeric form of the YadA head domain is essential for binding to collagen type I (Figure 3A). Mutation of the hydrophobic core to charged residues prevents collagen binding by disrupting the trimeric structure (Nummelin et al., 2004). YadA does not, however, recognize a specific collagen sequence, as has been shown by binding of YadA to different collagen-like peptides. The binding motif is structural, requiring the triple helical motif, but the sequences that bind *dis*favour Lys (Leo et al., 2008). The affinity of YadA for collagen peptides as measured by SPR (Surface Plasmon Resonance) is 0.3 μM (Nummelin et al., 2004). This was further confirmed by binding studies using collagen mimicking peptides and measured by SPR and ITC (Isothermal Titration Calorimetry). However, the triple collagen structure is not sufficient for tight binding and so the key to the tight affinity must be avidity: one YadA can recruit and bind multiple collagen molecules and vice versa. Despite the lack of a specific sequence requirement for binding, YadA most tightly interacts with iminoacids, especially 4-hydroxyproline rich regions that contain few charged residues (Leo et al., 2010).

However, on cells, the interaction of collagen fibrils with YadA is more complex. The collagen is most likely positioned on the head diagonally, at a crossing angle of about 30° as suggested by docking experiments performed *in silico* supplemented with the experimental binding data on a set of mutants (Nummelin et al., 2004). Consequently, bending of YadA has to occur for two reasons: first, to expose the YadA collagen-binding head, as YadA is hugely overexpressed upon infection (Hoiczyk et al., 2000) and second to avoid steric clashes between the collagen fibrils and the bacterial surface (Nummelin et al., 2004). Since the collagen fibril is much longer and rigid (10–300 nm in diameter) than the YadA stalk, bending of the latter must occur (Nummelin et al., 2004). The proposed model of bending of the coiled-coil stalk would expose the YadA on the surface of the bacteria not only for binding collagen but also for binding other extracellular molecules (Nummelin et al., 2004). The high density and bending properties of YadA expressed on bacterial cell increase its avidity for collagen.

YeYadA protein binds other ECM molecules, fibronectin and laminin, but with significantly lower affinity than collagen (Tertti et al., 1992; Flügel et al., 1994). Laminin and fibronection bind to different sequence and structural motifs of YadA than collagen (Flügel et al., 1994). YeYadA binds both laminin-1 and laminin-2. For the former, binding was identified after elastase digestion to be the E1 fragment (Flügel et al., 1994). Secondly, YeYadA binds cellular fibronectin with affinity independent of the solid phase used for immobilization (nitrocellulose filter, plastic, and glass) and, with low affinity, plasma fibronectin, but only when it is immobilized on glass (Schulze-Koops et al., 1993). The YadA interaction with cellular fibronectin is fairly stable and heat resistant (no effect on binding after heating 45 min at 60°C). YadA does not bind plasma fibronectin unless immobilized on glass, suggesting that glass-immobilization exposes the YadA binding site on fibronectin. Another possibility is that domains present in cellular, but not plasma, fibronectins play crucial role in binding to YadA (Schulze-Koops et al., 1993). What is more, binding of YadA to fibronectinis is not dependent on the RGDS motif (the classical binding site on fibronectin for integrins of eukaryotic cells) (Schulze-Koops et al., 1993). In contrast, *Y. pseudotuberculosis* YadA (YpYadA) preferentially binds fibronectin, but not collagen and laminin. This is because of the presence of an additional 31 amino acid residues in the head region (Heise and Dersch, 2006). This extension has been named an “uptake domain,” since its deletion (residues 53–3) reduce YpYadA-mediated cell invasion. A deletion mutant of YpYadA lacking this sequence gains the features of YeYadA; bacteria lose high affinity binding to fibronectin and gain collagen and laminin binding (Heise and Dersch, 2006), but the structural basis for this is unclear.

Furthermore, YadA mediates adhesion to various cell types, like epithelial cells, neutrophils and macrophages (Figure 3A) (Leo and Skurnik, 2011). YpYadA facilitates epithelial cell entry when *Y. pseudotuberculosis* lacks invasin activity. This is in contrast to YeYadA (Eitel and Dersch, 2002). Binding to epithelial cells occurs through β_1_ integrins. However, for YadA, binding does not occur directly, like for invasin, but by ECM bridging. YadA interacts with ECM molecules presented on epithelial cells, which triggers β_1_ integrins binding to ECM molecules. This leads to activation of signaling cascades, which initiate actin cytoskeleton rearrangements and IL-8 production (Eitel and Dersch, 2002). IL-8 production recruits leukocytes, and as YadA expressing bacteria are resistant to phagocytosis, this recruitment leads to tissue damage and further dissemination of the bacteria (Grassl et al., 2003).

Bacterial survival in the host requires not only resistance to the adaptive immune system, but also to the innate one. YadA plays a key role in blocking all three pathways of complement: classical, alternative, and lectin (Lambris et al., 2008). This is crucial for *Y. enterocolitica* endurance, but not for *Y. pseudotuberculosis* (El Tahir and Skurnik, 2001). YadA binds to the complement regulators factor H (FH) (China et al., 1994) and C4bp (Kirjavainen et al., 2008). It thus acts as a negative regulator for both the classical and alternative pathways. The FH recruited by YadA is believed to lead to the downregulation of C3b by acting as a cofactor for factor I, which cleaves C3b to iC3b, and also by causing dissociation of the C3bBb complex (Biedzka-Sarek et al., 2008a). FH-YadA binding is salt resistant: it was still detected at 650 mM salt (Biedzka-Sarek et al., 2008a). FH binding has been mapped to the YadA stalk domain (Figure 3A) (Biedzka-Sarek et al., 2008b). Deletions of overlapping segments in YadA stalk did not prevent FH binding to YadA, which indicates that no specific sequence in bound by FH but interactions seems to be dependent on several conformational and discontinuous structural motifs (Biedzka-Sarek et al., 2008b). Moreover, the binding site of YadA on FH is not localized on certain FH domains, but it appears to throughout the entire molecule (Biedzka-Sarek et al., 2008b). Similarly, binding of C4b by YadA protects bacteria from opsonization followed by phagocytosis and activation of serum. Binding between YadA and C4bp is direct and probably involves charged residues, as it is strongly dependent on ionic strength: it is half reduced at 100 mM salt concentration and abolished at 250 mM (Kirjavainen et al., 2008).

YadA is responsible for other activities (Table 1), including mediating autoagglutination. The head domain possesses self-affinity and interacts in an antiparallel zipper-like manner (the more distal part of the protein interacts) causing bacterial flocculation (Hoiczyk et al., 2000). Autoagglutination may help bacteria in avoiding host immunology system and remain longer in the host. Moreover, YadA can bind to intestinal mucus, mucin, and brush border vesicles from rabbits (Mantle et al., 1989). This may increase colonization of the gut, or just be a host defence mechanism that prevents the bacteria binding to epithelial cells (Mantle and Husar, 1993). Finally, it acts as a docking system for the type III injectosome for delivery of antiphagocytic Yop proteins into the cytoplasm of the professional phagocytes (Visser et al., 1995). The length of the YadA extracellular domain has to be adjusted to the length of injectisome (or vice versa) to allow the injectisome to come into contact with target cell membrane (Mota et al., 2005). The injectisome and YadA or another delivery component must therefore coevolve.

## YadB and YadC—novel adhesins belonging to the TAA family

*Y. pestis* has undergone significant gene loss in speciating from *Y. pseudotuberculosis*, and inactivated genes include those coding for YadA and Invasin (Rosqvist et al., 1988; Simonet et al., 1996). This may be a reflection of changes in the life style of *Y. pestis* in comparison with the EPEC *Y. pseudotuberculosis*. Instead, *Y. pestis* contains two chromosomally encoded orthologues of YadA, YadB, and YadC (Forman et al., 2008). The *yadB* and *yadC* genes are located in a bicistronic operon. This uncommon arrangement may suggest a functional relationship like creation of a complex on the bacterial cell surface (Forman et al., 2008). Expression of YadB and C, similarly to YadA, is maximal at 37°C in stationary phase (Forman et al., 2008).

YadB (35 kDa) and YadC (61.6 kDa) are analogous to YadA but share similarity only in the C-terminal part (14% identity and 20% similarity over 264 residues) and within a short neck domain (10 of 21 residues are identical) (Forman et al., 2008). There is also resemblance in the stalk periodicity between YadA and YadC. On the other hand, the head region of YadC has no sequence similarity to YadA or to any other protein, and the predicted repeats have different periodicity than that present in YadA. YadB appears to have only a small head domain (7.3 kDa) with 29% identity to the corresponding region in YadC (Forman et al., 2008).

YadB and YadC, similarly to YadA, can form oligomers leading to aggregation (Forman et al., 2008). They mediate invasion to epithelial cells, although are not necessary for adherence. Mutation of both proteins significantly decreases the ability of the bacteria to invade HeLa epithelioid cells and type I pneumocytes but does not affect their ability to adhere to the cells (Forman et al., 2008). In addition, deletion of YadB and YadC caused a 2000-fold increase in subcutaneous LD_50_ in a pneumonic plague mouse model (Forman et al., 2008); in comparison, deletion of PsaA (protein that builds pH 6 antigen; explained in details below) led to just a 100-fold increase in subcutaneous LD_50_ (Cathelyn et al., 2006). YadB and YadC are not required for *Y. pestis* virulence in pneumonic plague but are essential for virulence properties and full lethality in a mouse model of bubonic plague (Forman et al., 2008). YadB and C also occur in *Y. pseudotuberculosis*, though not in *Y. enterocolitica*, but they are not key proteins during infection (Forman et al., 2008). Further studies are thus needed to pin down the role of YadB and YadC.

## Ail—the poorly exposed adhesin

Ail (which stands for attachment-invasion locus) is a chromosomally encoded 17 kDa protein that is associated with *Yersinia* virulence (Miller and Falkow, 1988; Miller et al., 1990). Expression of Ail by bacteria in the stationary phase occurs exclusively at 37°C under reduced oxygen partial pressure and is affected by pH, whereas expression in the log-phase is detectable at lower temperatures (Pierson and Falkow, 1993).

**Figure 4:**
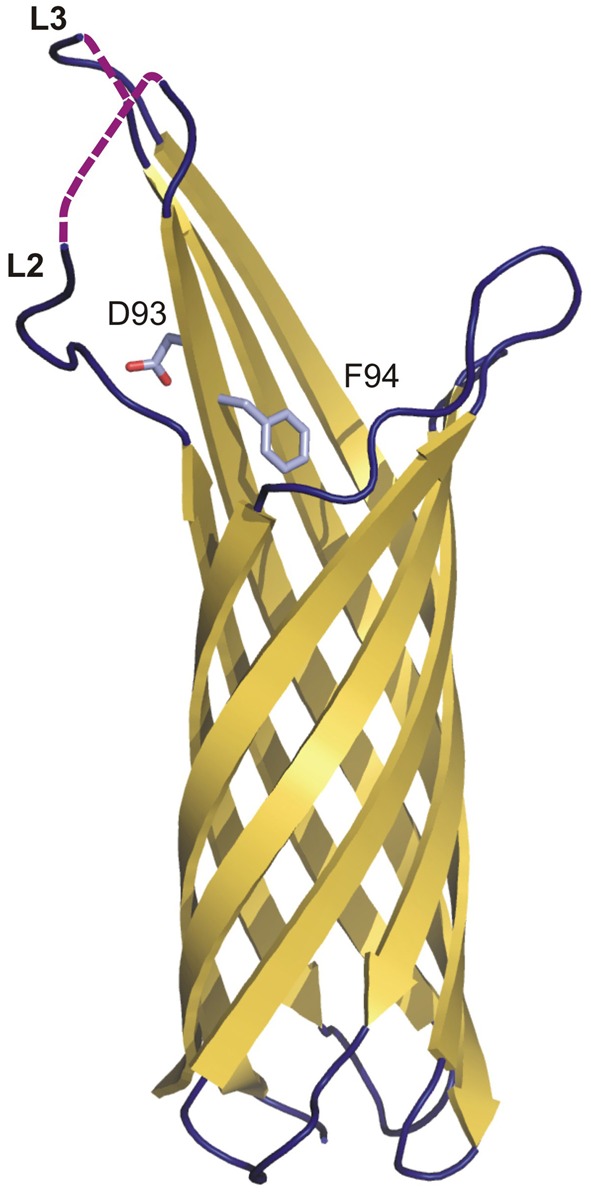
**The crystal structure of Ail from *Y. pestis*.** Topology diagram of the Ail structure (3QRA) with partially disordered loops two (L2) and three (L3). The invisible loop regions are marked on the picture as violet dashed lines. Residues D93 and F94 (crucial in serum resistance and invasion) are shown as sticks.

The structure of *Y. pestis* Ail has recently been determined (PDB: 3QRA) (Figure 4) (Yamashita et al., 2011). Ail forms an OM-embedded 8-stranded β-barrel structurally related to the *E. coli* invasion protein OmpX. The structure thus has the topology predicted earlier (Miller et al., 2001). The β strands vary in length between 10 and 18 residues, with the height of the barrel varying from 35 to 53 Å. The lumen of the barrel, which is filled with side chains, does not form a channel, and the barrel is elliptical in cross-section with axes of 12 × 20 Å. There are four extracellular loops that contribute to adhesion properties (Figure 4).

The individual residues that mediate adhesion in *Y. pestis* Ail have not been determined. However, the location of the residues conferring pathogenic activity has been identified in *Y. enterocolitica* Ail. They are in the extracellular loops 2 and 3, of which loop 2 is absolutely required for both invasion and serum resistance (Figure 4) (Miller et al., 2001). Mutational analysis demonstrated that residues responsible for the serum-resistance phenotype are located at the C-terminal end of loop 2 and the N-terminal end of loop 3, and those responsible only for the invasion phenotype are located in the middle of loop 2 and the C-terminal end of loop 3. D67 and V68 are the only residues for which mutation causes both loss of serum resistance and complete elimination of the invasion phenotype. They are located at the C-terminal end of loop 2. Loops 1 and 4 do not contain residues directly involved in the invasion or serum resistance phenotypes.

D67 and V68 from *Y. enterocolitica* correspond to D93 and F94 from *Y. pestis*, which are located on the fourth β-strand close to the bilayer interface (Fairman et al., 2012). In the structure, the D93 side chain protrudes outside the barrel to the extracellular space and F94 enters the barrel interior. *Y. enterocolitica* and *Y. pestis* Ails have limited sequence similarity and many of the residues identified in the former are not conserved in the latter, especially in loops 1, 2, and 3 (Kolodziejek et al., 2012). However, residues that have been shown to be important in pathogenicity in *Y. enterocolitica* Ail are clustered around a hydrophobic cleft in *Y. pestis* Ail.

Ail takes part in serum resistance in all three *Yersinia* pathogenic species. It binds factor H (FH) and C4 binding protein (C4bp), and so downregulates complement activation in the same way as YadA, leading to bacterial survival (Biedzka-Sarek et al., 2008b). It has been shown that Ail targets only single short consensus repeats in FH (Biedzka-Sarek et al., 2008a). However, due to its small size, it is usually masked by LPS O-antigen in *Y. enterocolitica* and *Y. pseudotuberculosis* (Figure 1A). Thus it plays an active role only if the bacteria have rough LPS (Biedzka-Sarek et al., 2005).

Adhesion to epithelial cells and ECM proteins is another important activity of *Y. enterocolitica* and *Y. pestis* Ail. *Y. pseudotuberculosis* Ail lacks the conserved residues responsible for binding to ECM (Miller et al., 2001). This also indicates that invasion and serum resistance are separated. The ECM components targeted by Ail are laminin, fibronectin and heparan sulfate proteoglycan (Yamashita et al., 2011). Binding to laminin and fibronectin plays a significant role in Yop delivery, as blocking of those proteins significantly reduced Yop distribution (Yamashita et al., 2011). Moreover, Yamashita et al. determined by elastase digestion mapping that the Ail binding site is on laminin G-like domains 4 and 5 (Yamashita et al., 2011). Ail is one of the main adhesins in *Y. pestis* responsible for Yop delivery through the type III secretion system, presumably by ensuring correct placement of the bacterial injectisome on the host cell. Unlike pH6 antigen and Pla (see below), it is functional both at 28 and 37°C (Felek et al., 2010).

## Pla—*Y. pestis* unique adhesin

Plasminogen activator (Pla) is a virulence factor, adhesin and protease (Felek et al., 2010) encoded on plasmid pPCP1 and highly expressed during host invasion by *Y. pestis*. It is crucial for establishment of bubonic plague in animals by maintaining metastasis from intradermal tissue to lymph nodes. Moreover, in pneumonic plague in humans, it allows *Y. pestis* to grow in airways (Haiko et al., 2009).

**Figure 5:**
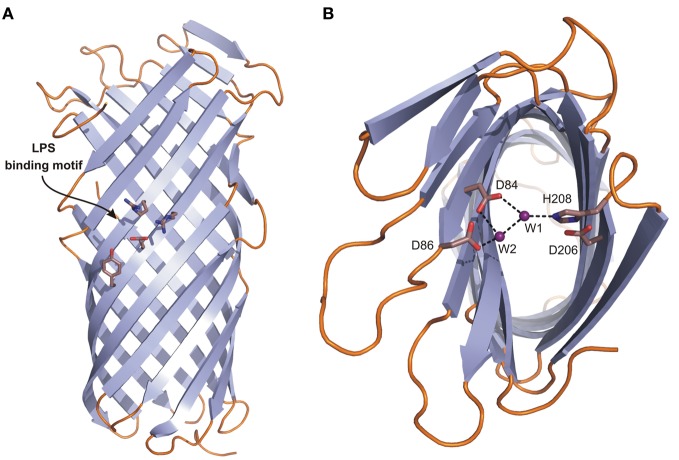
**The crystal structure of Pla from *Y. pestis*.** Topology diagrams of the Pla structure (2X55). **(A)** Side view presenting the position of the putative LPS binding site (residues D84, D86, D206, and H208 in stick representation). **(B)** Top view showing the active site. D84 and D86 coordinate nucleophilic water molecule W1 either directly or indirectly via second water molecule W2. D206 and H208, the latter being hydrogen bonded to W1, form a catalytic dyad.

Pla belongs to the family of OM proteases/adhesins known as omptins that share high sequence identity but differ in biological function (Kukkonen and Korhonen, 2004; Haiko et al., 2009). They are widely distributed in Gram-negative pathogenic bacteria infecting both animals and plants. The structure of Pla has a narrow β-barrel with an elliptical cross-section composed of 10 antiparallel β-strands from which five loops L1–L5 are exposed to the extracellular environmental milieu (PDB: 2X55) (Figures 5A,B) (Eren et al., 2010). The periplasmic side contains short turns only. The barrel is about 70 Å long at its highest point.

Omptins appear to constitute a unique class of proteases. Their conserved catalytic residues Asp84, Asp86, Asp206, and His208 (Pla numbering) differ from those found in other proteases suggesting a new catalytic mechanism, involving an Asp-His catalytic dyad and an Asp-Asp couple that activates the nucleophilic water molecule (Figure 5B) (Kramer et al., 2001; Vandeputte-Rutten et al., 2001; Baaden and Sansom, 2004). The presence of a catalytic dyad is unique to omptins among proteases, though examples exist in the α/β hydrolase fold family (Ollis et al., 1992; Heikinheimo et al., 1999). In Pla the active site is located in a cleft on the extracellular surface of the barrel (Figure 5A) (Eren et al., 2010). The pairs are located on opposite sites of the barrel with their side chains facing the lumen. The Asp84–Asp86 pair plays a supporting rather than a central role in catalysis (Figure 5B) (Kukkonen et al., 2001). It provides important structural support via coordination of the nucleophilic water molecule, either directly or indirectly via second water molecule W2 (Figure 5B) (Eren et al., 2010). This notion is supported by the pH-activity profiles showing that omptins are inactive below pH 5.0 (Kramer et al., 2000; Eren et al., 2010).

The second unique feature of omptins is that they require the presence of rough LPS for enzymatic activity and are inhibited by the O-antigen chains present in smooth LPS (Kramer et al., 2002; Kukkonen et al., 2004). The previously suggested LPS binding motif based on the structure of FhuA (the OM receptor for ferrichrome-iron in *E. coli*) (Ferguson et al., 1998) corresponds to the following Pla residues: Tyr134, Glu136, Arg138, and Arg171. Indeed, parallel tubes of electron density resembling LPS chains were located close to this site (Eren et al., 2010). A possible explanation of LPS-dependent activity is that, in the absence of LPS, Pla undergoes a subtle conformational change that widens the active site groove. This causes the substrate peptide to diffuse deeper into the active site, replacing the catalytic water molecules and rendering the enzyme inactive (Eren and van den Berg, 2012). Moreover, the functionality of Pla is affected by the level of acylation of lipid A, which is present in LPS. The level of acylation is pH and temperature dependent, and is low at 37°C. This increases the fluidity of the bacterial OM making Pla more active (Haiko et al., 2009).

The main function of Pla is to activate plasminogen to plasmin (Beesley et al., 1967; Degen et al., 2007). Plasmin degrades networks in extracellular matrices by activation of proMMPs (matrix metalloproteinases), which enables cell migration. The uncontrolled activation of plasmin by Pla, and at the same time inactivation of the antiprotease α_2_-antiplasmin (α_2_AP) (plasmin deactivator) allows *Y. pestis* to invade the host rapidly and to migrate to lymphatic tissue (Plow et al., 1999; Sebbane et al., 2006). Another activity of plasmin is degradation of laminin (the glycoprotein of the basement membranes), as well as fibrin clots, which are created just after injury of blood vessel walls. This activity also helps bacteria migrate faster, taking control of the human homeostatic system of coagulation/fibrinolysis and influencing inflammatory responses (Haiko et al., 2009). Moreover, Pla enables dissemination of *Y. pestis* from the initial infection site to other organs by interacting with CD205 present on macrophages. It mediates adherence and invasion into human epithelial cells and allows establishment of infection in liver and spleen (Sodeinde et al., 1992). Finally, Pla may play a role in serum resistance by interfering with the complement system (Sodeinde et al., 1992) as it cleaves complement protein C3, thus reducing opsonophagocytosis of infecting bacteria and preventing neutrophil recruitment to the site of infection (Sodeinde et al., 1992). On the other hand, Haiko et al. (2009) have shown that *Y. pestis* strains lacking Pla remain resistant to serum, again suggesting that this is not likely to be its main function.

Finally, Pla has been shown to contribute to cytotoxicity of *Y. pestis* because it, like Ail, is important in delivering Yop proteins to epithelial host cells (Felek et al., 2010) especially at 37°C. It is involved in positioning the type III secretion system so that the Yops can be injected and in mouse models, is a major contributor to *Y. pestis* virulence (Felek et al., 2010). In this function, it replaces YadA and invasin in *Y. enterocolitica* and *Y. pseudotuberculosis*.

## pH 6 antigen—acidic, antiphagocytic adhesin

pH 6 antigen is a putative adhesin associated with *Y. pestis* virulence. It has a homopolymeric fibrillar structure consisting of 15 kDa subunits of PsaA protein (Lindler et al., 1990). After expression of PsaA and its secretion into the periplasmic space via the Sec machinery, its transport through the periplasm is assisted by chaperones whose function is to prevent polymerization and mediate its transport to the OM (Soto and Hultgren, 1999).

As long as 1961, Ben-Efraim et al. showed that bacteria incubated at pH less 6 and 37°C had decreased electrophoretic mobility, due to expression of an antigenic component, and cells unable to express this component were attenuated by the host immunological system. They thus named it the pH 6 antigen (Ben-Efraim et al., 1961). This is true: pH 6 antigen is maximally expressed during the early stationary phase at pH between 5 and 6.7, at 37°C, but it is functional between pH 4 and 10 (Payne et al., 1998). Moreover, mutants of the pH 6 antigen gene *psaA* showed a 200-fold increase in the 50% lethal dose in comparison to wild type (Lindler et al., 1990). Expression of pH 6 antigen is thus crucial during bacterial infection of *Y. pestis*, which is consistent with findings showing that bacteria expressing *psaA* are resistant to phagocytosis (Huang and Lindler, 2004).

*Y. pestis* producing pH 6 antigen can agglutinate erythrocytes from a variety of species (Yang et al., 1996). pH 6 antigen expressed by *Y. pestis* inside phagolysosomes in macrophages (Lindler and Tall, 1993) may facilitate escape from them. pH 6 antigen may also protect bacteria against further phagocytosis (Makoveichuk et al., 2003) by interacting with apolipoprotein B (apoB)-containing lipoproteins in human plasma. The interactions are to the lipid moieties, usually in low-density lipoprotein (LDL). Bacteria coated with LDL would be protected against immunological recognition (Makoveichuk et al., 2003) by uptake prevention, and by blocking of adhesin-receptor interaction (Huang and Lindler, 2004). On the other hand, liposome binding can interfere or obstruct further infection steps (Makoveichuk et al., 2003). Binding to epithelial cells (Yang et al., 1996), macrophages and human fibroblasts is facilitated by interaction of pH 6 antigen with lipid rafts (Makoveichuk et al., 2003).

Additionally, pH 6 antigen binds also to β1-linked galactosyl residues in glycosphingolipids, which are found among different cell types (Payne et al., 1998). Finally, it protects *Y. pestis* against host recognition by binding human Fc of immunoglobulin G in a non-immune manner (Zav'yalov et al., 1996) analogous to EibD (Leo and Goldman, 2009) or protein G (Sjöbring et al., 1991). As with Ail and Pla, pH 6 antigen also facilitates Yop delivery by the type III secretion system, though it appears to be not as important in this as the other two (Felek et al., 2010).

*Y. pseudotuberculosis* and *Y. enterocolitica* also express pH 6 antigen, (the Myf, mucoid *Yersinia* factor) (Leo and Skurnik, 2011). Similarly to pH 6 antigen, it is expressed at 37°C, in acidic conditions and its synthesis depends on five chromosomal genes *myfA*, *B*, *C*, *E*, and *F* (Iriarte and Cornelis, 1995). The main product of the Myf antigen is the 14 kDa MyfA protein, which after polymerization creates a fibrous layer of Myf antigen. MyfA and pH 6 antigen both form fibrilar structures surrounding the bacteria. Immunogold labeling of MyfA revealed that it appears as a layer of extracellular material that extends two microns from the bacterial surface (Iriarte et al., 1993). Electron microscopy studies supplemented with immunogold labeling showed that pH 6 antigen forms flexible fibrillar organelles composed of individual linear strands, multiple strands or wiry aggregates composed of PsaA protein subunits (Lindler and Tall, 1993). It has been shown that Myf antigen mediates thermoinducible adhesion of *Y. pseudotuberculosis* to tissue culture cells, and similarly pH 6 antigen takes part in hemagglutination (Yang et al., 1996). In contrast, *Y. enterocolitica* MyfA does not promote hemagglutination and its function as an adhesin and involvement in phagocytosis is still not clear (Yang et al., 1996).

## Conclusions

The first Yersinia adhesin identified was pH 6 antigen, discovered in 1961 (Ben-Efraim et al., 1961), and since then molecular biology and structural biology have identified and catalogued a variety of adhesins, not only for *Yersinia* spp (e.g., YadA, Pla, Ail, Invasin), but also related adhesins in other bacteria species.

All the bacteria possessing genes for adhesins express them on the cell surface during the first stages of host infection. Usually, they comprise the majority of proteins present in the OM facing outside of the cell; YadA, UspA1 (from *Moraxella catarrhalis*), and BadA (from *Bartonella henselae*) are good examples of this. The adhesins play crucial roles in cell attachment, invasion, serum resistance and so in the maintenance and spread of the infection, by interacting with different cell types and host molecules. Even though we continually gain insight into their molecular mechanism, the exact role of adhesins during infection remains unclear. This is mainly because each bacterium expresses multiple adhesins that have overlapping functions and binding affinities and even after deletion of one adhesin another typically compensates for its function at least in part. Moreover, due to the complexity of bacteria–bacteria and bacteria–host interactions, exactly which adhesins are expressed during the various phases of infection has not been easy to establish: the *in vitro* experiments altering pH and temperature only crudely mimic the situation *in vivo*, when the bacteria experience shear stresses due to, e.g., blood flow and form biofilms. In this, the use of genuine tissue samples such as primary umbilical cord (Riess et al., 2004) has made it possible to study certain phenomena *ex vivo* under conditions close to *in vivo*. Finally, while the structure of many adhesins is known, there are no structures of adhesin-ligand complexes. That makes even more difficult to establish how they function.

To resolve the contribution of each adhesin in phatogenicity will require highly sensitive protein techniques to identify the cell-surface molecules expressed in small primary populations of bacteria taken directly from host tissue as well as *ex vivo* experiments. Understanding the interactions of adhesins with their ligands at the molecular level by solving structures between bacterial adhesins and host ligands, and using this information for drug and vaccine design, is the next major challenge.

### Conflict of interest statement

The authors declare that the research was conducted in the absence of any commercial or financial relationships that could be construed as a potential conflict of interest.
